# Radiolological predictors of recurrence of chronic subdural hematoma

**DOI:** 10.12669/pjms.341.13735

**Published:** 2018

**Authors:** Imran Altaf, Shahzad Shams, Anjum Habib Vohra

**Affiliations:** 1 Dr. Imran Altaf, MS. Department of Neurosurgery, Khawja Muhammad Safdar Medical College, Sialkot, Pakistan; 2 Dr. Shahzad Shams, FRCS, FCPS. Department of Neurosurgery, Post Graduate Medical Institute, Lahore General Hospital, Lahore, Pakistan; 3 Dr. Anjum Habib Vohra, FRCS. Department of Neurosurgery, Post Graduate Medical Institute, Lahore General Hospital, Lahore, Pakistan

**Keywords:** Chronic subdural hematoma, Recurrence, Radiological factors

## Abstract

**Objective:**

Chronic subdural hematoma is one of the most common clinical entities encountered in daily neurosurgical practice. Considerable recurrence rates have been reported for chronic subdural hematoma following surgical evacuation. Many studies have suggested various radiological factors that may be associated with the recurrence of CSDH. However, the results are inconsistent. This study focuses on determining the radiological factors predictive of chronic subdural hematoma recurrence.

**Methods:**

A retrospective analysis of 113 patients diagnosed with chronic subdural hematoma who were surgically treated between August 2013 and December 2014 was performed. The radiological features were analyzed to clarify the correlation between these radiological factors and postoperative recurrence of chronic subdural hematoma.

**Results:**

Twenty patients (17.7%) experienced recurrence. Chronic subdural hematoma recurrence was found to be significantly associated (p<0.05) with preoperative hematoma thickness ≥ 20 mm. Midline shift, hematoma density and bilaterality were not significantly associated with recurrence. Post operative drainage also significantly (p<0.05) reduced chronic subdural hematoma recurrence.

**Conclusion:**

Preoperative hematoma thickness ≥ 20 mm is an independent predictor of recurrence of chronic subdural hematoma. Postoperative drainage also significantly reduces chronic subdural hematoma recurrence.

## INTRODUCTION

Chronic subdural hematoma (CSDH) is one of the most common clinical entities encountered in daily neurosurgical practice.^1^-^5^ The incidence of CSDH has been reported to be as high as 13.1 cases per 100000 inhabitants.^6^ The condition is treated by various surgical procedures including burr-hole trephination, twist-drill trephination and craniotomy.^7^-^10^ Of these procedures, burrhole evacuation is the most popular technique worldwide.^1^,^6^,^7^ However, considerable recurrence rates have been reported ranging from 3 to 30% following surgical management.^7^,^9^,^11^,^12^

Many radiological criteria related to recurrence have been reviewed in literature including preoperative hematoma width, preoperative midline shift, hematoma density and bilateral CSDH.^6^,^7^,^9^,^13^ Studies conducted regarding correlation of these factors with recurrence have shown conflicting results, and no consensus exists on the subject.^6^,^10^,^13^-^20^ This study evaluated the correlation of these different radiological factors with recurrence.

## METHODS

The medical and radiographic records of 113 cases with CSDH who underwent surgery for CSDH in the department of neurosurgery Lahore General Hospital between August 2013 and December 2014 were retrospectively reviewed. Diagnosis was confirmed by CT (computed tomography) scan in all patients. All patients enrolled in the study had been treated with two burr holes with or without drainage, and had more than three months follow-up period. Patients treated with a single burr hole or craniotomy were excluded from the study. The drain if placed was removed after 48 hours. We defined CSDH as a SDH surrounded by a thin capsule and consisting of dark reddish liquefied blood found at operation. Postoperative CT scan was obtained within three days after surgery. Thereafter CT was requested if there was clinical deterioration or recurrence of symptoms. Recurrence of CSDH was defined as re-accumulation of blood within the postoperative hematoma cavity on follow up CT scan within the first three months after surgery and re-appearance of neurological symptoms.

The preoperative thickness of the hematoma was measured on CT scan, which revealed the maximal thickness of the hematoma. The degree of midline shift was measured near the level of the third ventricle or septum pellucidum on CT scan. Hematoma type was classified into five types according to their density on CT scan: hyperdense, isodense, hypodense, mixed-density and the trabecular stage which featured septation of walls.

The patients were divided into two groups according to the recurrence of CSDH. The clinical and radiological factors were compared between the recurrence group and the no recurrence group.

Statistical analysis was performed with independent t-test for sex, and chi-square test for other parameters to assess their relationship with recurrence of CSDH. For all analysis, a *p*-value of <0.05 was considered statistically significant.

### Ethics committee approval

As the study is a retrospective study with no interventions so ethics committee approval was not required.

## RESULTS

There were 113 patients included in the study. The baseline characteristics of the patients with CSDH area shown in Table-I. There were 91 males (80.5%) and 22 females (19.5%) in the study, ranging in age from 40 to 90 years (mean age, 63.45 years). Twenty patients (17.7%) experienced recurrence. There were 18 males (90%) and two females (10%) in the recurrence group (RG), ranging in age from 41 to 81 years (mean age, 59.35 years). Mean age of patients in the recurrence group (RG) (59.35) was not significantly different from that in the non- recurrence group (NRG) (64.33). Twenty eight patients had bilateral hematoma. In eight of these 28 patients both sides were operated. Overall, in 113 patients 121 sides were operated for chronic SDH.

**Table-I:** Demographic characteristics of patients in the recurred group and the group with no recurrence.

	RG (n= 20)	NRG(n= 93)	Total	p-value
Sex				0.238
Male	18 (90%)	73(78.5%)	91 (80.5%)
Female	2 (10%)	20 (21.5%)	22 (19.5%)
Age Mean ± SD (years)	59.35 ±11.95	64.33±11.11	63.45±11.37	0.075

RG= Recurrence group NRG=Non-recurrence group.

Table-II Shows the relationship between different preoperative radiological factors and recurrence. Recurrence was found to be significantly associated (p<0.05) with preoperative hematoma thickness. Midline shift, hematoma density and bilaterality were not found to be significantly associated with recurrence. The association of post-operative drainage with recurrence is shown in Table-III.

**Table-II:** Preoperative radiologic features of chronic subdural hematoma on brain computed tomography.

Radiologic features	Number of patients (%)			p-value

	RG	NRG	Total	
*Thickness*				
< 20 mm	2 (10%)	44 (43.6%)	46 (38%)	0.005
≥ 20 mm	18 (90%)	57 (56.4%)	75 (62%)	
*Density*				
Iso	0	27 (26.7%)	27 (22.3%)	0.097
Low	2 (10%)	12 (11.9%)	14 (11.6%)	
High	2 (10%)	10 (9.9%)	12 (9.9%)	
Mixed	12 (60%)	38 (37.6%)	50 (41.3%)	
Septated	4 (20%)	14 (13.9%)	18 (14.9%)	
*Midline Shift (Unilateral lesion)*				
< 10 mm	0	10 (3.7%)	10 (11.8%)	0.172
≥ 10 mm	12 (100%)	63 (86.3%)	75 (88.2%)	
*Laterality*				
Unilateral	12 (60%)	73 (78.5%)	85 (75.2%)	0.082
Bilateral	8 (40%)	20 (21.5%)	28 (24.8%)	

**Table-III:** Comparison of recurrence rates with and without drainage.

	RG (n= 20)	NRG(n= 101)	Total	p-value
Drain	4(20%)	59(58.4%)	63(52.1%)	0.002
No Drain	16(80%)	42(41.6%)	58(47.9%)	

## DISCUSSION

CSDH is a common disease in neurosurgical practice and the incidence is increasing.^6^. The reported recurrence rate ranges from 3% to 30% where this study showed a recurrence rate of 17.7%. The etiology of CSDH recurrence has not been completely understood until now,^6^ but several radiological risk factors for CSDH recurrence have been reported, including: hematoma thickness, hematoma density, bilateral CSDHs and preoperative midline shift. However, results identifying consistent risk factors have been difficult to reproduce.^6^,^7^,^9^,^10^,^13^-^20^ In this study the authors sought to identify radiological risk factors for CSDH recurrence requiring repeat drainage.

### Hematoma thickness

Several studies have identified greater hematoma thickness as a risk factor for recurrence.^12^,^18^,^20^ Yamamoto et al.^21^ proposed that larger hematomas present greater tendency of recurrence since subdural space following surgical evacuation is larger than in smaller hematomas. The authors also found large hematomas ≥20mm to be significantly correlated with higher recurrence rates.

### Midline shift

Preoperative midline displacement as a predictor of recurrence is debatable, with studies showing conflicting results. Jung Min Lee et al.^6^ and Dae Hyo Song et al.^15^ found preoperative midline displacement to be an independent predictor of CSDH recurrence, whereas Ecosa Bae M et al.^10^ and Yoon-Gyo Jung et al.^17^ concluded that preoperative midline displacement was not significantly associated with recurrence. In our study the association between recurrence and preoperative midline shift was not found to be statistically significant.

### Density

Hematoma density has also been cited by many authors as a risk factor for recurrence.^12^,^20^,^22^,^23^

In our study, however, statistically significant association was not found between the hematoma density and recurrence. Our findings are thus consistent with the findings of Jung Min Lee et al.^6^ and Yoon-Gyo Jung et al.^17^ who did not find an association between hematoma density and recurrence.

### Bilateral hematomas

Theoretically, patients with bilateral CSDH tend to have previous brain atrophy, which may lead to poor brain re-expansion.^18^ A few previous studies propose higher recurrence rates in bilateral CSDH.^6^,^14^,^15^ This correlation was not, however, statistically significant in our study. However, even with statistical insignificance, bilateral CSDH could present rapid and progressive aggravation with increased intracranial pressure, and thus, surgical treatment should be considered earlier if indicated.^12^

### Post operative drain

The efficacy of postoperative drainage after CSDH is a controversial topic. Some prospective studies showed no beneficial effect, whereas other authors report lower recurrence rates with the use of postoperative drains because of brain expansion.^3^,^8^,^22^,^24^,^25^ A recent meta-analysis concluded that postoperative drainage is clearly useful in the treatment of CSDH and should be recommended.^2^ Our findings also corobrate this conclusion as post operative drainage significantly (p<0.05) reduced the CSDH recurrence rate in our study.

### Limitations of the study

This is a small size retrospective and non-randomized study. Therefore, it is potentially subject to diverse biases and variations. Further investigation with larger sample size, quantitative controlled prospective study is required to clarify the definite radiological risk factors for recurrence of CSDH.

## CONCLUSION

Preoperative hematoma thickness ≥ 20 mm is an independent predictor of recurrence of chronic subdural hematoma. Postoperative drainage also significantly reduces chronic subdural hematoma recurrence.

### Authors' Contribution

***IA*** conceived, designed, did data collection & statistical analysis & manuscript writing & editing of manuscript

***SS & AHV*** did review and final approval of manuscript.
